# Equity in children’s dental caries before and after cessation of community water fluoridation: differential impact by dental insurance status and geographic material deprivation

**DOI:** 10.1186/s12939-016-0312-1

**Published:** 2016-02-11

**Authors:** Lindsay McLaren, Deborah A. McNeil, Melissa Potestio, Steve Patterson, Salima Thawer, Peter Faris, Congshi Shi, Luke Shwart

**Affiliations:** Department of Community Health Sciences, University of Calgary, Calgary, AB Canada; Research and Innovation, Population, Public and Aboriginal Health, Alberta Health Services, Calgary, AB Canada; Alberta Cancer Prevention Legacy Fund, Population Public and Aboriginal Health, Alberta Health Services, Calgary, AB Canada; Faculty of Medicine and Dentistry, School of Dentistry, University of Alberta, Edmonton, AB Canada; Research Facilitation, Priorities, and Implementation, Alberta Health Services, Calgary, AB Canada; Provincial Oral Health Office, Population, Public and Aboriginal Health, Alberta Health Services, Calgary, AB Canada

**Keywords:** Fluoridation, Dental health surveys, Health equity, Public health dentistry, Socioeconomic factors

## Abstract

**Background:**

One of the main arguments made in favor of community water fluoridation is that it is equitable in its impact on dental caries (i.e., helps to offset inequities in dental caries). Although an equitable effect of fluoridation has been demonstrated in cross-sectional studies, it has not been studied in the context of cessation of community water fluoridation (CWF). The objective of this study was to compare the socio-economic patterns of children’s dental caries (tooth decay) in Calgary, Canada, in 2009/10 when CWF was in place, and in 2013/14, after it had been discontinued.

**Methods:**

We analyzed data from population-based samples of schoolchildren (grade 2) in 2009/10 and 2013/14. Data on dental caries (decayed, missing, and filled primary and permanent teeth) were gathered via open mouth exams conducted in schools by registered dental hygienists. We examined the association between dental caries and 1) presence/absence of dental insurance and 2) small area index of material deprivation, using Poisson (zero-inflated) and logistic regression, for both time points separately. For small-area material deprivation at each time point, we also computed the concentration index of inequality for each outcome variable.

**Results:**

Statistically significant inequities by dental insurance status and by small area material deprivation were more apparent in 2013/14 than in 2009/10.

**Conclusions:**

Results are consistent with increasing inequities in dental caries following cessation of CWF. However, further research is needed to 1) confirm the effects in a study that includes a comparison community, and 2) explore possible alternative reasons for the findings, including changes in treatment and preventive programming.

## Background

There are significant socio-economic inequities in dental caries (tooth decay). Data from Canada [1], the U.S. [2], Australia [3], the United Kingdom [4], Brazil [5] and elsewhere [6] have demonstrated more, and more severe, dental caries among children experiencing one or more of: low family income, lower household educational attainment, racial/ethnic minority status, lack of private dental insurance, and higher levels of deprivation based on small area measures. In some cases, inequities were shown to be increasing (worsening) over time [5, 7].

Dental caries is largely preventable, and community water fluoridation (CWF) (i.e., the controlled addition of a fluoride compound to a public water supply [8]) is one important option for prevention at the population level. Extensive research supports the benefits of CWF for the prevention of tooth decay in populations, although the overall methodological quality of the evidence is modest, due to problems such as weak study designs that inadequately account for potential confounders [9, 10]. A recent Cochrane review concluded that high-quality recent evidence was particularly sparse [10].

One of the main arguments made in favor of CWF is that it is equitable in its impact on dental caries [11]. In other words, it has been argued that CWF can help offset inequities in dental caries, by benefiting all but particularly those experiencing lower socioeconomic circumstances, who generally have the poorest oral health profiles. An equitable effect of CWF has been demonstrated in cross-sectional studies in several countries, including Canada [12], Britain [13], Australia [14], New Zealand [15] and South Korea [16]. For example, Jones and Worthington [13] showed that a positive linear association between dental caries (decayed, missing, and filled deciduous teeth) and small area deprivation score (based on the Townsend index) was much steeper in non-fluoridated Liverpool, UK, than in Newcastle where CWF had been in place since the 1960s. Recently, Cho et al. [16], in a study of 11-year old children in 8 geographic areas in South Korea (4 fluoridated areas and 4 non-fluoridated areas which were similar in economic situation, population size, and geography), showed a statistically significant negative association between socioeconomic status (family affluence scale) and DMFT index (decayed, missing, or filled permanent teeth) in the non-fluoridated areas, but no association in the fluoridated areas. The multivariable analysis adjusted for intake of cariogenic snacks and beverages, oral hygiene behaviors (brushing and flossing), and use of piped water for drinking and cooking.

An equitable effect is consistent with CWF as a population-level intervention that is structural in nature [17, 18]. That is, rather than acting on individuals’ behaviors, it acts on the circumstances in which behaviors occur, and thus the issue of active uptake-which is often inequitably patterned-is largely obviated [18]. Research on population-level interventions and their implications for health equity is very important to the field of population/public health, which is concerned with understanding and reducing social inequities in health [19–21]. Social inequities in health refer to differences in health status between social groups that are viewed as unfair and avoidable, as distinct from inequalities in health which refer simply to differences without accompanying moral and ethical dimensions [22]. Research on fluoridation and social inequities in dental health, thus, makes an important contribution to that broader field of inquiry.

Although an equitable effect of CWF has been shown in cross-sectional studies, equity of impact has not been investigated in the context of cessation (i.e., do inequities in dental caries increase [worsen] after community water fluoridation is stopped?). To illustrate, in a systematic review of research on cessation of CWF [McLaren L, Singhal S. Does cessation of community water fluoridation lead to an increase in tooth decay? A systematic review of published studies. Unpublished], we identified published research on cessation in 15 jurisdictions across 13 countries. None of these incorporated an analysis of equity of impact.

The objective of this paper was to explore the equity implications of CWF cessation, by examining socio-economic patterns of children’s dental caries in Calgary, Canada, in 2009/10 when CWF was in place, and in 2013/14, after it had been discontinued. This paper is part of a larger project, the objective of which was to evaluate the short-term impact of CWF cessation on children’s dental caries by comparing Calgary (where CWF was discontinued in May 2011, after having been in place since 1991) to Edmonton (where CWF began in 1967 and remains in place). Elsewhere [McLaren L, Patterson S, Thawer S, Faris P, McNeil D, Potestio M, Shwart L. The short-term impact ofcommunity water fluoridation cessation on children’s dental caries: a natural experiment in Alberta, Canada. Unpublished; 23], we reported main effects results from that comparative evaluation. In those papers, we were not able to examine questions about equity because socio-economic data were not available in our pre-cessation (2004/05) surveys for both cities.

Here we use data from another Calgary survey, from 2009/10 [24], which contained some socio-economic information, to explore trends over time by socio-economic indicators. The specific objective was to examine change over time in children’s dental caries in Calgary by 1) presence or absence of dental insurance, and 2) a geographic small area measure-namely, material deprivation of child’s community of residence (dissemination area). Based on consistent findings from cross-sectional studies, we hypothesized that we would see an increase in inequity of dental caries, by dental insurance and small area material deprivation, post-cessation relative to pre-cessation.

## Methods

### Data source

Data for a population-based sample of grade 2 students in Calgary in 2009/10 were available from a survey conducted by the former Calgary Health Region as part of their surveillance activities [24]. Data for a population-based sample of grade 2 students in Calgary in 2013/14 were collected as part of a joint research - surveillance initiative to evaluate the impact of CWF cessation in Calgary on children’s dental caries [25]. Methods used in 2013/14 were designed to maximize comparability with the earlier survey. In both surveys, dental caries data (decayed, missing/extracted, and filled teeth) were collected via open mouth exams conducted in schools, by trained and calibrated assessment teams each consisting of a registered dental hygienist and clerk.

The target population for both surveys was children (grade 2) attending school in the Public or Catholic school systems in the city of Calgary. In both survey years (2009/10 and 2013/14) a stratified random sample was drawn, with strata based on median neighbourhood income where the school was located; within sampled schools, all children of eligible grades were invited to participate. Signed parental informed consent was secured, and verbal assent of each child was also secured. The response rate in 2009/10 was 81 % [24]. In 2013/14 the response rate was lower: the overall school-level response rate for Calgary was 57 %, and the overall student-level response rate within participating schools was 49 %. We developed sampling weights for both surveys. The weights account for both the probability of selection and the probability of non-response, and enhance the extent to which each sample (2009/10 and 2013/14) is representative of its underlying target population. We also ran all analyses unweighted. Results were broadly similar in all cases (i.e., no change to statistical significance vs. not) and weighted estimates are presented below.

### Variables and analysis

Outcome variables examined were: the deft index and the DMFT index. These indices were created by summing, for each child, the number of decayed, extracted/missing (due to caries) or filled teeth for both primary teeth (deft) and permanent teeth (DMFT), based on the open mouth exam. We focus on tooth-level measures (versus tooth surface-level measures) because the 2009/10 survey only contains tooth-level data. The deft and DMFT are well-established, commonly used summary measures for studies of tooth decay in child populations [26]. Because the deft and DMFT include both treated (fillings, extractions) and untreated decay, which could show different socio-economic patterns depending on access to treatment [27], we also examined untreated decay separately. Specifically, we considered those with two or more teeth (primary or permanent) with untreated decay (“decay2”) as a dichotomous variable (yes vs. no).

Two socioeconomic indicators were considered, based on information available in both surveys. The information was included on the parent questionnaire in 2013/14, and on the parent consent form in 2009/10. The first indicator was reported presence vs. absence of dental insurance, where presence included any type of insurance (i.e., private, employer-sponsored, or public). Second, we used the Pampalon index, which describes the material deprivation of a small geographic area [28]. The Pampalon index is based on age- and sex-adjusted census data from Statistics Canada (from the 2006 census^1^) for the population age 15 years and older within a dissemination area. A dissemination area is a geographic unit used in the Canadian census that contains a population of 400–700 persons. It is the smallest standard geographic area for which aggregate census data are released [29]. Material deprivation is a composite variable based on: average individual income, employment to population ratio, and proportion without a high school diploma or equivalent. The Pampalon index was assigned to survey respondents by linking each respondent’s home postal code to the corresponding dissemination area. In the 2009/10 survey, school postal code was recorded if the home postal code was not provided; we omitted cases in which that occurred (*n* = 43 cases, approx. 8 % of the sample). Material deprivation was expressed as a continuous variable based on factor analysis. Using the continuous variable scores for the full Alberta population, we created both quintiles and tertiles which were then applied to our sample, for use in regression analysis below.

We first examined the association between socio-economic indicators and dental caries measures for 2009/10 and 2013/14 separately, using zero-inflated Poisson regression (deft, DMFT) or logistic regression (decay2). We then confirmed apparent differences between surveys using zero-inflated Poisson regression or logistic regression including a year X socio-economic indicator interaction term (where X = multiplied by), for each socio-economic indicator and each dental caries outcome measure separately.

Then, for material deprivation, we computed the concentration index of inequality [30] for each dental caries outcome variable. The concentration index, which can range from −1 to +1, quantifies the extent to which a health problem is concentrated in lower (or higher) socio-economic groups. Means and regression coefficients do not necessarily capture this information. For the concentration index we used the continuous version of the Pampalon material deprivation index, because the concentration index requires socio-economic data that are at least at the ordinal level of measurement. The continuous variable was transformed so that a negative concentration index, as per convention, corresponds to greater concentration of the health problem among those with fewer resources (higher deprivation). To improve the meaning of the concentration index, one can multiply the value by 75 which yields the percentage of the health variable that would need to be redistributed from the less deprived half to the more deprived half of the population (if the inequality favors the less deprived) to arrive at an equal distribution (i.e., a concentration index of 0) [30].

The study received approval from the Conjoint Health Research Ethics Board at the University of Calgary (ID E-25219) and the Health Research Ethics Board at the University of Alberta (ID Pro00037808). Approval was also sought and granted by the participating school boards.

## Results

Full sample sizes for primary teeth were: *n* = 557 (2009/10) and *n* = 3230 (2013/14). Sample sizes for permanent teeth were slightly lower because those measures are based on individuals with at least one permanent tooth: *n* = 551 (2009/10) and *n* = 3182 (2013/14). Sample sizes for analyses with dental insurance were approximately 98–99 % of the full sample, due to missing data on dental insurance: *n* = 528 (deft) and *n* = 522 (DMFT) for 2009/10, and *n* = 3164 (deft) and *n* = 3120 (DMFT) for 2013/14. For the small-area material deprivation index, sample size was approximately 92 % of the full sample, partly because the deprivation index is based on 2006 census data which omits some newer postal codes: *n* = 511 (deft) and *n* = 505 (DMFT) for 2009/10, and *n* = 2980 (deft) and *n* = 2939 (DMFT) for 2013/14.

Means or percentages and 95 % confidence intervals for all study variables are shown in Table 1, for 2009/10 and 2013/14 separately.

**Table 1:** Descriptive statistics for study samples (weighted estimates)

Variable	Calgary 2009/10:	Calgary 2013/14:
	Mean or % (95 % CI), *n*	Mean or % (95 % CI), *n*
Dental caries summary measures		
Mean number of decayed, extracted, or filled primary teeth (deft)	2.22 (1.87 to 2.57), *n* = 557	2.69 (2.52 to 2.86), *n* = 3230
Mean number of deft among those with deft > 0	4.22 (3.85 to 4.58), *n* = 284	4.73 (4.52 to 4.94), *n* = 1835
Mean number of decayed, missing, or filled permanent teeth (DMFT)	0.19 (0.11 to 0.27), *n* = 551	0.12 (0.10 to 0.14), *n* = 3182
Mean DMFT among those with DMFT > 0	1.85 (1.61 to 2.09), *n* = 56	1.52 (1.41 to 1.63), *n* = 254
Percent with 2 or more teeth with untreated decay (primary or permanent)	10 % (8 % to 13 %), *n* = 551	14 % (12 % to 15 %), *n* = 3182
Socio-economic variables		
Percent with no dental insurance	21 % (17 % to 25 %), *n* = 528	17 % (15 % to 19 %), *n* = 3164
Small area material deprivation (Pampalon index)^a^		
Percent within Category 1 (least deprived)	39 % (27 % to 52 %)	34 % (29 % to 40 %)
Percent within Category 2	18 % (12 % to 27 %)	23 % (18 % to 28 %)
Percent within Category 3	16 % (11 % to 23 %)	15 % (11 % to 19 %)
Percent within Category 4	9 % (6 % to 12 %)	11 % (9 % to 13 %)
Percent within Category 5 (most deprived)	19 % (10 % to 31 %)	18 % (14 % to 23 %)
	*n* = 511	*n* = 2980

^a^Pampalon material deprivation categories are based on quintiles that apply to the whole province of Alberta. Category 1 = least deprived, 5 = most deprived.

Tables 2 and 3 shows associations between socio-economic variables and dental caries summary measures in 2009/10 and 2013/14 based on regression analyses. Table 2 focuses on dental insurance. Absence of dental insurance was associated with higher mean DMFT in 2013/14 but not in 2009/10. A statistically significant year X no dental insurance interaction term was observed for DMFT (far right-hand column of Table 2), indicating that the association between no dental insurance and DMFT differed significantly between 2009/10 and 2013/14. Absence of dental insurance was associated with greater likelihood of having two or more instances of untreated decay, in both 2009/10 and 2013/14.

**Table 2:** Weighted estimates from regression (zero-inflated Poisson, logistic) to assess associations between dental insurance (no vs. yes) and dental caries indices, Grade 2 students in Calgary, 2009–10 and 2013/14

Outcome variable	Rate ratio (RR) or odds ratio (OR) for effect of absence (vs presence) of dental insurance on dental caries outcomes (reference = 1.0)		
	2009/10	2013/14	Interaction term (Year X No dental insurance): RR or OR (95 % CI), *p*-value, (*n*)
	RR or OR (95 % CI), *p*-value, (*n*)	RR or OR (95 % CI), *p*-value, (*n*)	
deft^a^	RR = 1.05 (0.94 to 1.17), *p* = 0.40 (*n* = 528)	RR = 0.94 (0.86 to 1.03), *p* = 0.18 (*n* = 3164)	RR = 0.90 (0.78 to 1.04), *p* = .14 (*n* = 3692)
DMFT^a^	RR = 0.87 (0.65 to 1.16), *p* = 0.33 (*n* = 522)	RR = 1.56 (1.05 to 2.33), *p* = 0.03^*^ (*n* = 3120)	RR = 1.80 (1.10 to 2.93), *p* = .02^*^ (*n* = 3642)
2 or more teeth (primary or permanent) with untreated decay^b^	OR = 1.76 (1.34 to 2.32), *p* < .001^*^ (*n* = 522)	OR = 2.0 (1.57 to 2.53), *p* < .001^*^ (*n* = 3120)	OR = 1.13 (0.81 to 1.58), *p* = .46 (*n* = 3642)

*deft* number of decayed, missing, and filled primary teeth, *DMFT* number of decayed, missing, and filled permanent teeth, *X* multiplied by

^*^Statistically significant effect of no dental insurance (vs. dental insurance) on dental caries outcome

^a^Zero-inflated Poisson regression

^b^Logistic regression (yes vs. no)

**Table 3:** Weighted estimates from regression (zero-inflated poisson, logistic) to assess associations between Pampalon material deprivation index categories corresponding to provincial tertiles (highest deprivation and middle deprivation versus lowest deprivation) and oral health summary measures, grade 2 students in Calgary, 2009–10 and 2013/14

Outcome variable	Rate ratio or odds ratio for effect of high or middle material deprivation (vs. low deprivation) on dental caries outcomes (reference = 1.0)		
	2009/10	2013/14	Interaction terms: RR or OR (95 % CI), *p*-value (*n*)
	RR or OR (95 % CI), *p*-value (*n*)	RR or OR (95 % CI), *p*-value (*n*)	
deft^a^	Highest deprivation: RR = 1.07	Highest deprivation: RR = 1.19	Year X Highest deprivation: RR = 1.11
	(0.93 to 1.23), *p* = .34	(1.08 to 1.30), *p* < .001^*^	(0.95 to 1.30), *p* = .20
	Middle deprivation: RR = 1.03	Middle deprivation: RR = 1.15	Year X Middle deprivation: RR = 1.12
	(0.88 to 1.19), *p* = .73 (*n* = 511)	(1.02 to 1.30), *p* = .023^*^ (*n* = 2980)	(0.91 to 1.37), *p* = .27 (*n* = 3491)
DMFT^a^	Highest deprivation: RR = 1.42	Highest deprivation: RR = 1.04	Year X Highest deprivation: RR = 0.74
	(.74 to 2.69), *p* = .27	(0.68 to 1.59), *p* = 0.85	(0.36 to 1.50), *p* = 0.40
	Middle deprivation: RR = 1.08	Middle deprivation: RR = 0.80	Year X Middle deprivation: RR = 0.74
	(0.61 to 1.91), *p* = .77 (*n* = 505)	(0.49 to 1.30), *p* = 0.37 (*n* = 2939)	(0.36 to 1.51), *p* = 0.41 (*n* = 3444)
2 or more teeth (permanent or primary) with untreated decay^b^	Highest deprivation: OR = 2.95	Highest deprivation: OR = 2.23	Year X Highest deprivation: OR = 0.75
	(0.89 to 9.82), *p* = .07	(1.66 to 2.98), *p* < .001^*^	(0.24 to 2.36), *p* = 0.63
	Middle deprivation: OR = 0.90	Middle deprivation: OR = 1.43	Year X Middle deprivation: OR = 1.59
	(0.31 to 2.62), *p* = .83 (*n* = 505)	(1.05 to 1.94), *p* = .024^*^ (*n* = 2939)	(0.57 to 4.43), *p* = 0.37 (*n* = 3444)

*deft* number of decayed, missing, and filled primary teeth, *DMFT* number of decayed, missing, and filled permanent teeth, *X* multiplied by

^*^Statistically significant effect (at *p* < .05) of Pampalon material deprivation category (high or middle, versus low) on dental caries outcome

^a^Zero-inflated Poisson regression

^b^Logistic regression (yes vs. no)

Table 3 shows the associations between small area (dissemination area-level) material deprivation (Pampalon index) and dental caries summary measures. For primary tooth decay, there were statistically significant positive effects of material deprivation category (where categories correspond to provincial tertiles) with highest and middle material deprivation categories having higher [worse] primary tooth caries, relative to lowest material deprivation category, in 2013/14 but not in 2009/10. Highest and middle material deprivation, relative to lowest material deprivation, had greater likelihood of two or more instances of untreated decay in 2013/14 (positive effects at *p* < .05). In 2009/10 a positive effect of highest material deprivation came close to statistical significance (*p* = .07). There were no statistically significant year X material deprivation interactions for material deprivation.

**Fig. 1:**
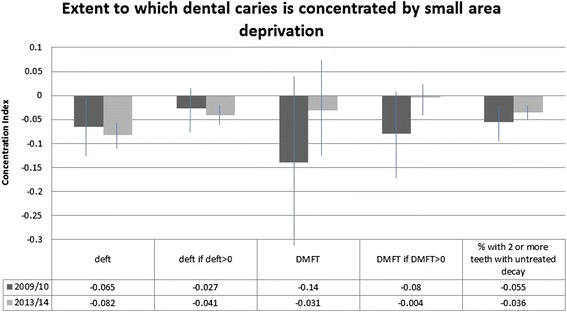
Extent to which dental caries is concentrated by small area deprivation. deft = number of decayed, missing, and filled primary teeth. DMFT = number of decayed, missing, and filled permanent teeth

Concentration indices showing the extent to which dental caries measures were concentrated across material deprivation (Pampalon index) are shown in Table 4, and illustrated in Fig. 1. In 2013/14, the primary tooth measures (mean deft, mean deft if deft > 0) and the % with two or more instances of untreated decay showed statistically significant concentration among those living in communities with higher material deprivation. In 2009/10, the deft (but not deft if deft > 0) and the % with two or more instances of untreated decay showed statistically significant concentration with increasing material deprivation, with an effect that came close to statistical significance (*p* = .067) for DMFT if DMFT > 0. As noted above, one can multiply the value of the concentration index by 75 to yield the percentage of the health variable that would need to be redistributed from the less deprived half to the more deprived half of the population to arrive at an equal distribution (i.e., a concentration index of 0) [30]. The percentages of the outcome variable that would need to be distributed from the less deprived half to the more deprived half of the population, computed for those measures with a concentration index that was statistically significant at *p* < .10 (for this purpose only, we included the effects that came close to statistical significance at the *p* < .05 level), ranged from a low of 2.7 % (2013/14 decay2) to a high of 6.2 % (2013/14 deft).

**Table 4:** Concentration indices for dental caries indices, by small-area material deprivation (Pampalon index)

Outcome variable	Concentration index which indicates the extent to which the dental caries index is concentrated by small-area deprivation^a^	
	2009/10	2013/14
	Concentration index (95 % confidence interval), *p*-value, (*n*)	Concentration index (95 % confidence interval), *p*-value, (*n*)
deft	Conc. index: −0.065 (−0.13 to −003), *p* = .039^*^	Conc. index: −0.082 (−0.11 to −0.06), *p* < .001^*^
	(*n* = 511)	(*n* = 2980)
deft if deft > 0	Conc. index: −0.027 (−0.07 to 0.017), *p* = .23	Conc. index: −0.041 (−.06 to −02), *p* < .001^*^
	(*n* = 256)	(*n* = 1678)
DMFT	Conc. index: −0.14 (−0.31 to 0.04), *p* = .13	Conc. index: −0.031 (−0.12 to 0.055), *p* = .48
	(*n* = 505)	(*n* = 2939)
DMFT if DMFT > 0	Conc. index: −0.08 (−0.17 to .006), *p* = .067	Conc. index: −0.004 (−.042 to 0.034), *p* = .84
	(*n* = 48)	(*n* = 237)
2 or more teeth (permanent or primary) with untreated decay	Conc. index: −0.055 (−0.09 to −0.019), *p* = .003^*^	Conc. index: −0.036 (−.048 to −024), *p* < .001^*^
	(*n* = 505)	(*n* = 2939)

*deft* number of decayed, missing, and filled primary teeth, *DMFT* number of decayed, missing, and filled permanent teeth

^*^Statistically significant at *p* < .05

^a^Concentration index is bounded by −1 (all problems concentrated in the lowest SES) and +1 (all problems concentrated in the highest SES). Concentration index of zero = perfect equality. Here, a statistically significant negative concentration index indicates that the dental caries outcome is significantly concentrated amongst those with higher material deprivation

## Discussion

We set out to explore whether inequities in tooth-level measures of dental caries increased (worsened) following cessation of CWF, by examining the association of caries measures with socio-economic indicators (dental insurance and small area material deprivation [Pampalon index]) in Calgary in 2009/10 and in 2013/14. CWF cessation occurred in 2011. This analysis was based on an argument often made in favor of CWF; namely, that it can help offset inequities in dental health [11] and thus inequities could hypothetically emerge or worsen following cessation.

Overall, we observed more evidence of inequities in 2013/14 (post-cessation) than in 2009/10 (pre-cessation). While inequities by dental insurance status and small area material deprivation were observed in both 2009/10 and 2013/14, they occurred more frequently, across more outcome measures, in 2013/14 than in 2009/10. However in some cases, the statistically significant inequities observed in 2013/14 did not significantly differ from effects in 2009/10. Furthermore, the magnitude of the inequities, especially as quantified using the concentration index, was quite small in most cases.

In the absence of a comparison community, it is important to consider other factors that might explain an apparent increase in dental caries inequities from pre- to post- fluoridation cessation. One possibility is changes to the composition of the population over time. Although Calgary’s population has increased (from approximately 1.07 million in 2010 to approximately 1.20 million in 2014), the proportion of 5–14 year olds (the age range that includes our target population) has stayed the same (11.5 % for both time points) (Calgary Civic Census). In terms of socio-economic characteristics, our study data presented in Table 1 showed that there has been no clear shift in proportions across the provincial material deprivation categories over the time period (the categories correspond to quintiles developed for the province of Alberta as a whole, so comparisons are meaningful). Unfortunately, we did not have data on other important population attributes at both time points, either from the Calgary civic census or from our data (the 2009/10 survey did not include a questionnaire). And, it is possible that the absolute material circumstances within the deprivation categories has changed (to become more or less inequitable), and we were not able to capture that.

A second possibility relates to changes over time in publically-funded preventive dental programming. For school-aged children (including Grade 2) in Calgary, there is a targeted program of fluoride varnish application which is administered by the provincial (and formerly regional) health authority, and is delivered in schools by dental public health professionals. The program is targeted to schools located in communities characterized as having higher care needs based on household income data for that community [31]. There have been some changes to the programming during the last several years. Specifically, prior to 2008/09, children within targeted schools were invited to receive the fluoride varnish only if they had not received treatment at a dentist office within the previous 6 months or if their family did not have dental insurance. Since 2008/09, all children within targeted schools have been invited to receive the fluoride varnish. An additional change that occurred after CWF cessation in 2011 was that some funds were re-allocated to a dental health bus, which travels to schools in lower-income communities (which represent a subset of the schools targeted in the school-based program) to deliver services, including fluoride varnish and application of protective dental sealants.

Collectively, therefore, preventive programming that is delivered and funded by the provincial health authority for school-aged children in Calgary has-if anything-expanded over the time period, which is not consistent with it explaining our observed trend of an apparent increasing presence of dental caries inequities over the time period. The observation that inequities have increased despite these programs, which are targeted to socioeconomically disadvantaged populations, in fact raises interesting questions about the role of targeted, versus more universal, preventive programming vis-à-vis population dental health and health inequities [32]. Such questions are particularly pertinent as more communities opt to discontinue CWF [33], which represents a universal or population-level approach to prevention [17]. Discontinuation of CWF demands consideration of viable, effective, and equitable alternatives.

A third possible reason for an observed increase in dental caries inequities is inequities in dental-related behaviors such as regular tooth brushing with fluoride toothpaste. A post hoc exploration of our 2013/14 data (which included a questionnaire) indicated statistically significant inequities, by dental insurance status and by small area material deprivation (Pampalon index), in 1) reported brushing at least twice/day and 2) reported use of fluoride toothpaste. Those without dental insurance, and those living in areas characterized by higher material deprivation, were less likely to report brushing at least twice/day and less likely to report using fluoride toothpaste, compared to those with insurance, and those living in less deprived areas, respectively (more detailed analysis of socioeconomic inequities in dental-related behaviors in 2013/14 is planned for another paper). Thus, although our 2013/14 data indicate the presence of inequities in these behaviors, the lack of such information in 2009/10 precluded insight into whether or to what extent they may have contributed to apparently increasing inequities in dental outcomes observed here.

A fourth issue that must be considered, as possibly contributing to an apparent increase in dental caries inequities from pre- to post- fluoridation cessation, is dental care in the private sector. A recent report [27] highlighted the significant inequities in access to dental care across Canada, which reflects the overwhelmingly private nature of dental service delivery: of all oral health care expenditures in Canada, approximately 95 % is privately financed [27]. Theoretically, increasing inequities in dental caries following fluoridation cessation could reflect coincident trends toward less access to care, or greater inequities in access to care, during the time period in question. However, that does not seem likely in this case, for three reasons. Firstly, although the effect is not statistically significant, Table 1 hinted at a decline, if anything, in the proportions of our samples who did not have dental insurance. On the other hand, if changes to dental insurance have occurred such that those who responded “yes” had less coverage in 2013/14 than was the case in 2009/10, then insurance might still play a role and we would not have captured it here.

Furthermore, in another paper from the same project [McLaren L, Patterson S, Thawer S, Faris P, McNeil D, Potestio M, Shwart L. The short-term impact of community water fluoridation cessation on children’s dental caries: a natural experiment in Alberta, Canada. Unpublished], we showed that the proportion of Calgary children in our study with “complete caries care”, defined as one or more fillings or extractions but no untreated decay, had increased over time, while the proportion with “no caries care” (some untreated decay, but no fillings or extractions) had decreased over time. The opposite was observed in our comparison community of Edmonton. This suggested that care access has, if anything, increased in Calgary.

Finally, the fact that we observed emerging inequities in the composite caries measures (deft, DMFT, which include both treated and untreated problems), as well as in the indicator of untreated decay (which would reflect primary prevention rather than treatment), suggests that the factors driving the increasing inequity include factors that drive incidence. Overall, it seems unlikely that the factors contributing to the observed increase in inequities over time are predominantly dental care-related. Rather, the observed trends in inequities appear to have occurred despite hints of improvements in treatment over time.

A primary limitation of our study was the lack of comparison community for this analysis; however, the fact that we had a comparison community for the main analysis [McLaren L, Patterson S, Thawer S, Faris P, McNeil D, Potestio M, Shwart L. The short-term impact of community water fluoridation cessation on children’s dental caries: a natural experiment in Alberta, Canada. Unpublished; 23] permitted us to triangulate from those findings to explore consistency of effects. In the absence of a comparison community, a longitudinal design where the same children are studied at multiple time points would have been a superior design, which would have permitted better control for potential confounding factors. Another important limitation was that we had only very limited socio-economic information available at both time points. The dental insurance variable was crude, and the “yes” category included those with private or employer-sponsored insurance, as well as those with public insurance. To explore whether the conflation of different types of insurance influenced our findings, we ran a post hoc re-analysis of the 2013/14 data (for which we had information on type of insurance) in Table 2, omitting those with public insurance (who represented approximately 10 % of the sample). The pattern of findings did not change (i.e., statistically significant positive effect of no insurance on DMFT and on decay2). The material deprivation index (Pampalon index), while carefully constructed [28], is an ecological level variable and thus our understanding of the relationship between individual-level socio-economic characteristics and dental caries in the context of CWF cessation remains limited.

Strengths of our study included high-quality caries data collected by dental health professionals who underwent intensive and ongoing training and calibration led by a public health dentist with expertise and experience in survey calibration; population-based samples with sampling weights to permit better representation of the target population; and our pre-post CWF cessation approach which builds importantly on the existing (cross-sectional) literature of CWF and dental health inequities.

## Conclusions

Our study objective was to compare the socio-economic patterns of children’s dental caries in Calgary, Canada, before and after cessation of community water fluoridation. Overall, results are consistent with increasing inequities in dental caries following cessation of CWF. However, additional studies are needed to confirm the effects using a study design that permits stronger control for potential confounding variables, such as a pre-post design with comparison community, or a prospective longitudinal design where the same children are examined at multiple time points. Future research on the implications of CWF cessation for inequities in dental health outcomes should also ensure high-quality individual-level data on socio-economic circumstances both pre- and post- cessation.

## Abbreviations

CWF: community water fluoridation
deft: decayed, extracted (due to caries), and filled primary teeth
DMFT: decayed, missing (due to caries), and filled permanent teeth

## Footnotes

1 Following the 2006 national census, the mandatory long-form census was discontinued, and its replacement (the National Household Survey) was voluntary and may have some bias (http://www12.statcan.gc.ca/census-recensement/fc-rf/reports-rapports/r2_table-tableau_3-eng.cfm).

## References

[1] Canada H (2010). Report on the findings of the oral health component of the Canadian health measures survey, 2007–2009.

[2] Edelstein BL, Chinn CH (2009). Update on disparities in oral health and access to dental care for America’s children. Acad Pediat.

[3] Do LG, Spencer AJ, Slade GD, Ha DH, Roberts-Thomson KF, Liu P (2010). Trend of income-related inequality of child oral health in Australia. J Dent Res.

[4] Broomhead T, Baker SR, Jones K, Richardson A, Marshman Z (2014). What are the most accurate predictors of caries in children aged 5 years in the UK?. Community Dent Health.

[5] Kramer PF, Chaffee BW, Bertelli AE, Ferreira SH, Béria JU, Feldens CA (2014). Gains in children’s dental health differ by socioeconomic position: evidence of widening inequalities in southern Brazil. Int J Paediatr Dent.

[6] Schwendicke F, Dörfer CE, Schlattmann P, Page LF, Thomson WM, Paris S (2015). Socioeconomic inequality and caries: a systematic review and meta-analysis. J Dent Res.

[7] Armfield JM, Spencer AJ, Slade GD (2009). Changing inequalities in the distribution of caries associated with improving child oral health in Australia. J Public Health Dent.

[8] Burt BA, Eklund SA (1999). Dentistry, dental practice, and the community.

[9] McDonagh M, Whiting P, Bradley M, Cooper J, Sutton A, Chestnutt I, et al. A systematic review of public water fluoridation. York: NHS Centre for Reviews and Dissemination. University of York; 2000.

[10] Iheozor-Ejiofor Z, Worthington HV, Walsh T, O’Malley L, Clarkson JE, Macey R, et al. Water fluoridation for the prevention of dental caries (Review). Cochrane Library. 2015. doi:10.1002/14651858.CD010856.pub2. 10.1002/14651858.CD010856.pub2 PMC6953324 26092033

[11] Burt BA (2002). Fluoridation and social equity. J Public Health Dent.

[12] McLaren L, Emery JCH (2012). Drinking water fluoridation and oral health inequalities in Canadian children. Can J Public Health.

[13] Jones CM, Worthington H (2000). Water fluoridation, poverty and tooth decay in 12-year-old children. J Dent.

[14] Slade GD, Spencer AJ, Davies MJ, Stewart JF (1996). Influence of exposure to fluoridated water on socioeconomic inequalities in children’s caries experience. Community Dent Oral Epidemiol.

[15] Treasure ET, Dever JG (1994). Relationship of caries with socioeconomic status in 14-year-old children from communities with different fluoride histories. Community Dent Oral Epidemiol.

[16] Cho H-J, Lee H-S, Paik D-I, Bae K-H (2014). Association of dental caries with socioeconomic status in relation to different water fluoridation levels. Community Dent Oral Epidemiol.

[17] Rose G (1992). The strategy of preventive medicine.

[18] McLaren L, McIntyre L, Kirkpatrick S (2010). Rose’s population strategy of prevention need not increase social inequalities in health. Int J Epidemiol.

[19] Commission on Social Determinants of Health (CSDH) (2008). Closing the gap in a generation: health equity through action on the social determinants of health.

[20] Raphael D (2011). A discourse analysis of the social determinants of health. Crit Public Health.

[21] Mackenbach JP, Kulhánová I, Menvielle G, Bopp M, Borrell C, Costa G (2015). Trends in inequalities in premature mortality: a study of 3.2 million deaths in 13 European countries. J Epidemiol Community Health.

[22] Whitehead M (1991). The concepts and principles of equity and health. Health Promotion Int.

[23] McLaren L, Patterson S, Thawer S, Faris P, McNeil D, Potestio M, Shwart L. Measuring the short-term impact of fluoridation cessation on dental caries in Grade 2 children using tooth surface indices. In press, Community Dent Oral Epidemiol. 10.1111/cdoe.12215 PMC5021129 26888380

[24] Shwart L, Rodine L (2010). Children’s oral health survey, 2009–10.

[25] McLaren L, Shwart L, Faris P, McNeil D, McIntyre L, Patterson S, Potestio M. Fluoridation discontinuation in Calgary: a natural experiment to identify implications for child oral health and oral health equity. Canadian Institutes of Health Research (operating grant), 2013–2015.

[26] World Health Organization (WHO) (2013). Oral health surveys: basic methods.

[27] Canadian Academy of Health Sciences (CAHS) (2014). Improving access to oral health care for vulnerable people living in Canada.

[28] Pampalon R, Hamel D, Gamache P, Raymond G (2009). A deprivation index for health planning in Canada. Chron Dis Can.

[29] Statistics Canada. Census Dictionary. Available online at: http://www12.statcan.gc.ca/census-recensement/2011/ref/dict/indexeng.cfm.

[30] O’Donnell O, van Doorslaer E, Wagstaff A, Liondelow M (2008). Analyzing health equity using household survey data: a guide to techniques and their implementation.

[31] Alberta Health Services (2010). Oral health action plan 2010–2012.

[32] Carey G, Crammond B (2014). A glossary of policy frameworks: the many forms of ‘universalism’ and policy ‘targeting’. J Epidemiol Community Health.

[33] Journal of the Canadian Dental Association (JCDA) (2013). Understanding public decision-making on community water fluoridation. J Can Dent Assoc.

